# Intraosseous Vascular Access through the Anterior Mandible – A Cadaver Model Pilot Study

**DOI:** 10.1371/journal.pone.0112686

**Published:** 2014-11-18

**Authors:** Christin Goldschalt, Sara Doll, Brit Ihle, Joachim Kirsch, Till Sebastian Mutzbauer

**Affiliations:** 1 University of Heidelberg, Institute for Anatomy and Cell Biology, Heidelberg, Germany; 2 Mutzbauer&Partner, Maxillofacial Surgery and Anesthesiology, Zuerich, Switzerland; Erasmus Medical Centre, The Netherlands

## Abstract

**Background:**

Several insertion sites have been described for intraosseous puncture in cases of emergencies when a conventional vascular access cannot be established. This pilot study has been designed to evaluate the feasibility of the mandibular bone for the use of an intraosseous vascular access in a cadaver model.

**Methodology/Principal Findings:**

17 dentistry and 16 medical students participating in a voluntary course received a short introduction into the method and subsequently used the battery powered EZ-IO system with a 15 mm cannula for a puncture of the anterior mandible in 33 cadavers. The time needed to perform each procedure was evaluated. India ink was injected into the accesses and during the anatomy course cadavers were dissected to retrace the success or failure of the puncture. Dental students needed 25.5±18.9(mean±standard deviation)s and medical students 33±20.4 s for the procedure (p = 0.18). Floor of mouth extravasation occurred in both groups in 3 cases. Success rates were 82 and 75% (p = 0.93).

**Conclusions/Significance:**

Despite floor of mouth extravasation of injected fluid into a mandibular intraosseous access might severely complicate this procedure, the anterior mandible may be helpful as an alternative to other intraosseous and intravenous insertion sites when these are not available in medical emergencies.

## Introduction

In medical emergencies it has been recommended to choose an intraosseous route when an intravenous access cannot be established within a reasonable amount of time [1].

Several insertion sites may be used for intraosseous injection. The medial tibia [2] as well as the radius, [3] the humerus [4] and the manubrium sterni [5] have been used in medical emergencies.

The available approved medical devices range from manual cannulas [6], [7] and semi-automatic systems to battery powered drilling devices [8]–[10].

Even the skull has been evaluated as a possible intraosseous access site [11].

The manubrium sterni may be preferred in cases when the limbs are not accessible in cases such as burns [12] blast injuries, entrapment or combat trauma [13]. In rare cases, even the manubrium may not be available for puncture.

In dentistry intraosseous local anesthesia is a well established alternative local anesthetic technique [14]. Several drilling devices have been developed to perform a puncture of the jaw bone. Some of these devices can be attached to a conventional dental handpiece [15]. Injection is either performed directly through the drilling cannula or – in certain systems – through the cannula after removal of an integrated trocar [16].

This pilot study was designed to evaluate whether the anterior mandible may be successfully punctured to be used as emergency intraosseous vascular access. The question to answer was: Can an intraosseous infusion into the marrow space of the mandible access the vascular system?

The specific objective was a staining of vessels in the vicinity of the jaws after a successful puncture of the mandible by an automatic intraosseous drilling set. Furthermore, complication such as floor of mouth extravasation should be documented.

## Materials and Methods

### Study design

**Figure 1:**
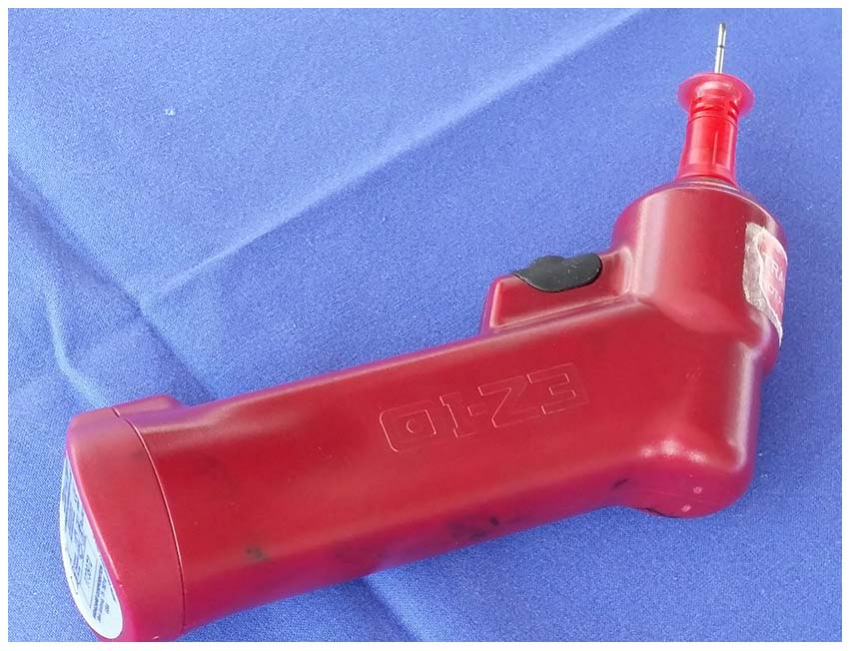
Motor driven battery powerd intraosseous drill device (equipped with 15mm needle).

Student participants of a voluntary vascular access training course in the dissecting room performed intraosseous punctures using a conventional battery driven intraosseous access drill device (figure 1) in cadavers embalmed for the regular student gross anatomy course.

### Study participants

Participating study subjects were medical and dental school students entering the course by signing into a list of limited spaces offered by the institute for anatomy each year. A waiting list enabled further students to participate, if occupied spaces were not taken at the date of the course. The numbers of students eligible for the course was mainly determined by the number of available cadavers. Prior to the study, none of the participants had been involved in intraosseous access application. Written informed consent was obtained from all participants. None of the investigators was involved in the rating of the students in running or future courses during medical and dental school training.

### Materials

The punctures were performed on adult human cadavers that were scheduled for dissection within the students' regular gross anatomy course. The cadavers had been embalmed by using a fixation process involving perfusion of isopropyl alcohol, Lysoformin (Lysoform, Berlin, Germany), formaldehyde, and glycerol via the femoral artery. Ethical approval included the consent that had been obtained from the body donors during their lifetime when they had enrolled into the willed body donor program of the university. The approval was issued by the ethics committee of the medical faculty of the university. The body donors had provided their written informed consent to participate in scientific evaluations and anatomy dissection courses, but not in this specific study, as the study had been planned after the body donors'death. Written agreement had been obtained during their lifetime.

Participant consent was documented within the body donor data base. The ethics committee had approved this consent procedure.

The sample size had primarily been determined by the number of available cadavers in the course. As the evaluation was designed as a pilot study, it was also intended to use obtained data for sample size calculations.

The semi-automatic battery driven drill device (EZ-IO, Vidacare, San Antonio TX, USA) equipped with the short 15 mm needle was used for puncture.

### Study procedure

**Figure 2:**
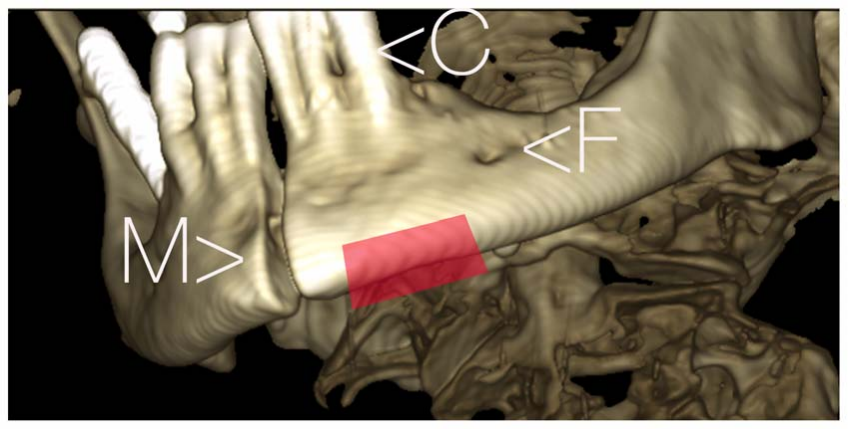
Area of the left lower jaw where the puncture should start at the bone level. The marked red area defines the borders within the procedure should be performed to avoid injury of the mental nerve. The position of the canine tooth C may facilitate choosing the drilling direction. M: midline F: foramen mentale.

**Figure 3:**
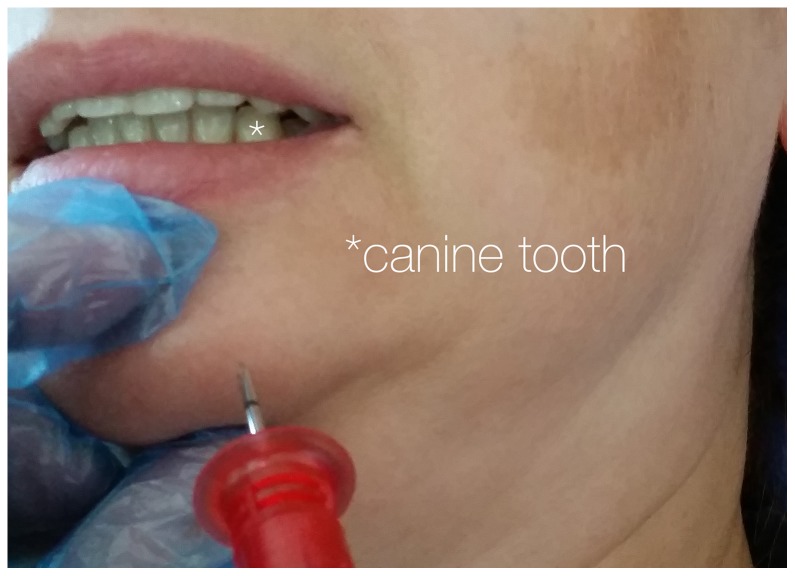
Position of the needle tip shown at a living female. Note, that the thumb and index finger stabilize the anterior lower jaw and facilitate the estimation of a correct drilling direction which is approximately 45 degrees towards the assumed root axis of the lower front teeth. The canine tooth is marked with *.

The application of the intraosseous cannula by use of the EZ-IO device for a tibia access was instructed by a 2 minute video demonstrating the handling of the device on the tibia. Subjects were then instructed to perform the puncture on the left side of the lower jaw of the test cadaver between midline and the assumed area of the foramen mentale (figures 2–3), which is always located in the area of the premolar roots, so choosing the area between midline and the canine tooth for the drilling procedure can avoid injury of the mental nerve. The drilling direction had to be chosen towards the suspected inner cortical layer of the lower jaw with an angle in the parasagittal plane of approximately 45 degrees towards the assumed root axis of the lower front teeth (Video S1). They should stop drilling after having placed the cannula tip within the anterior and posterior cortical layer of the mandible.

To avoid learning from success or failure from other procedures only one participant was allowed to enter the dissection room during each test. To familiarize the test persons with the intraosseous drill system they used it once immediately before mandibular puncture at the cadaver tibia, but did not receive a feedback about success or failure.

### Primary outcome measure

Once the IO needle had been inserted into the mandibula a 10 ml luer lock syringe containing india ink was connected. 1 ml of ink was injected into the cannula by a supervisor. Injection was stopped earlier if no resistance was detected and/or the ink emerged from the skin entrance hole. Earlier experiments had shown that even amounts of 0.1–0.2 ml of india ink have the capability of a very intense staining of vessels and tissue. Therefore, it was assumed that extravasation may be detected by the injection of very small amounts of india ink.

The body donors underwent the dissection course after the test. The individual puncture was rated as successful if india ink traces could be found in any peripheral vessels near the lower jaw of either skin, mucosa or tongue or in remote vessels such as the anterior jugular vein or – in one case – intracranial vessels. Floor of mouth extravasation was counted as a failure of the procedure even if a staining of vessels occurred.

### Secondary outcome measure

The time needed to perform the puncture needed by each participant was noted.

### Statistical analysis

Analyse-it 2.30 statistical software was used for data analysis. Puncture success and failure were noted in an Excel sheet generating two independent samples. Data were arranged in a 2×2 contingency table where each cell contained counts classified into the two participant groups and into success or failure.

A p-value lower than 0.05 indicated that the null hypothesis, the samples have the same proportion failure/success in each group, can be rejected.

The time each participant needed for puncture was entered into an Excel column. The collected data of both groups were then compared by a Mann-Whitney test for independent samples. p<0.05 allowed to reject the null hypothesis of equality of both distributions.

## Results

A total of 33 students participated. The dental student group consisted of 17, the medical student group of 16 individuals. The mandibles of 33 cadavers of the gross anatomy course were punctured.

### Demographic data

The medical students were in the preclinical part of their education, whereas the dental school students had just started with the clinical part.

### Puncture success (Table 1)

**Table 1:** Dental and medical students performing intraosseous vascular access by use of battery driven drill system.

n	33		
	team		
success	dent	med	Total
yes	14	12	26
no	3	4	7
Total	17	16	33
Difference of proportions	0.110		
95% CI	−0.304	to 0.524	(normal approximation)
2-tailed p	0.9266	(exact, double 1-tailed p)	

Success and failure of the procedure.

In the dental student group 14 and in the medical student group 12 punctures were rated as success. No differences were found comparing both groups (p = 0.93).

**Figure 4:**
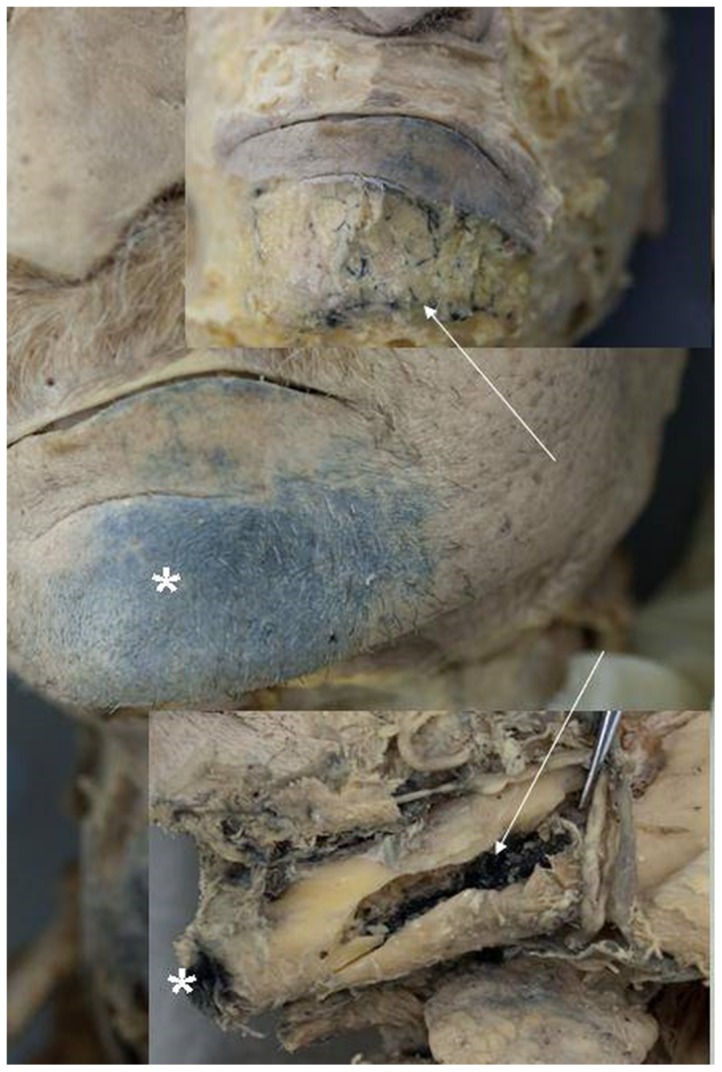
Buccal ink extravasation (asterisks) after intraosseous puncture of the left anterior mandible approximately half way between midline and the suspected area of the formamen mentale. In the upper figure inlet a vessel staining indication vascular deposition of ink is demonstrated. The arrows point to stained vessels.

In the dental group 3 puncture attempts resulted in an anterior subcutaneous extravasation of ink. In all of these cases ink could be detected in vessels. In all of the 6 cases in the medical student group where anterior subcutaneous ink extravasation was observed, ink was also detected in vessels (figure 4).

**Figure 5:**
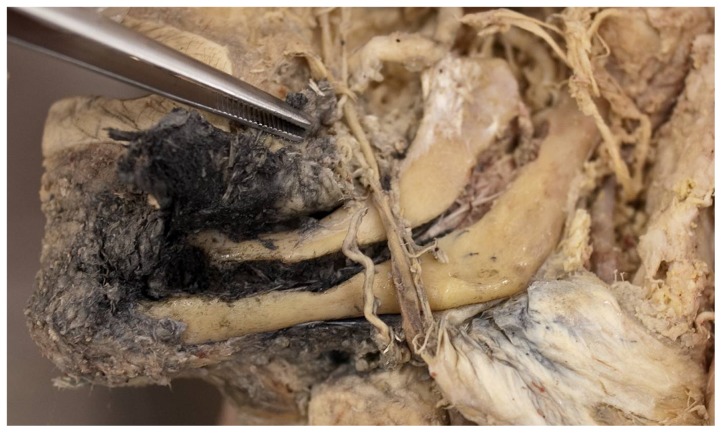
Floor of mouth extravasation of ink after injection through an intraosseos puncture needle placed in the left anterior mandible area. Similar staining of vessels in the inferior alveolar canal of the mandible.

3 puncture attempts ending in the floor of mouth soft tissue were observed in each group (figure 5). These attempts were classified as failures.

In the medical student group all specimens where a floor of mouth extravasation has been detected had also staining of the vessels.

### Time needed to perform the procedure (Figure 6)

**Figure 6:**
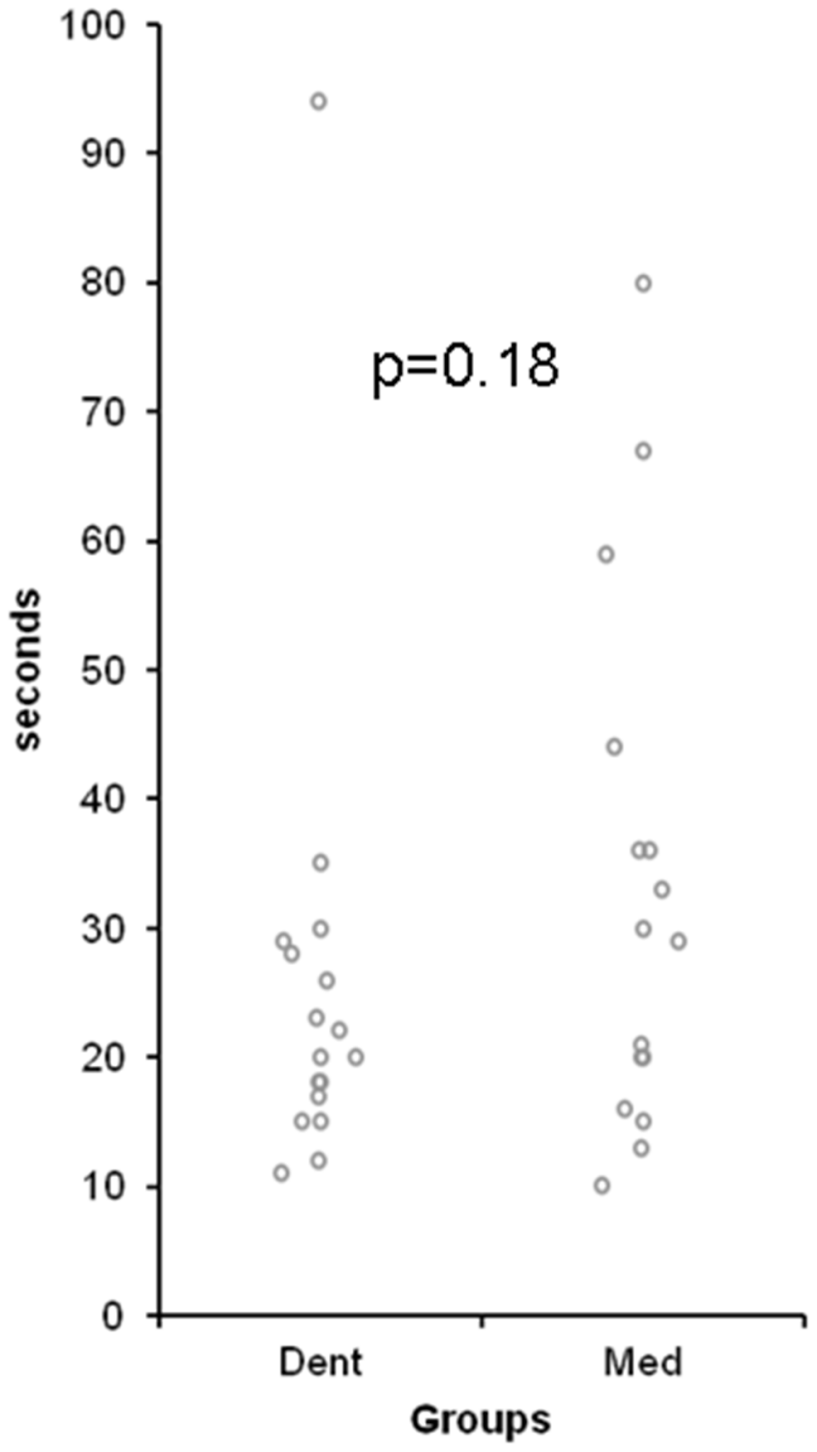
Time needed to perform an intraosseous vascular access using a battery driven intraosseous drill device. Dent – dental students n = 17, Med – medical students n = 16.

Dental students needed 25.5±18.9 (mean±standard deviation) seconds and medical students 33±20.4 seconds for the procedure. Comparing both groups regarding the times needed for puncture, no differences were found (p = 0.18).

## Discussion

This study is considered a feasibility study to determine whether or not the mandible is a possible intraosseous infusion site for clinical use in humans.

The intraosseous access as such is an emergency procedure that is not frequently used even by skilled emergency medical providers. For dentists, as a medical subpopulation, it may be even less likely to encounter situations that make such a procedure necessary.

To the best of our knowledge the mandibula has not been used for intraosseous infusion of emergency medication. However, intraosseous administration of local anaesthetics is a well known but also less common application technique for special considerations [17].

**Figure 7:**
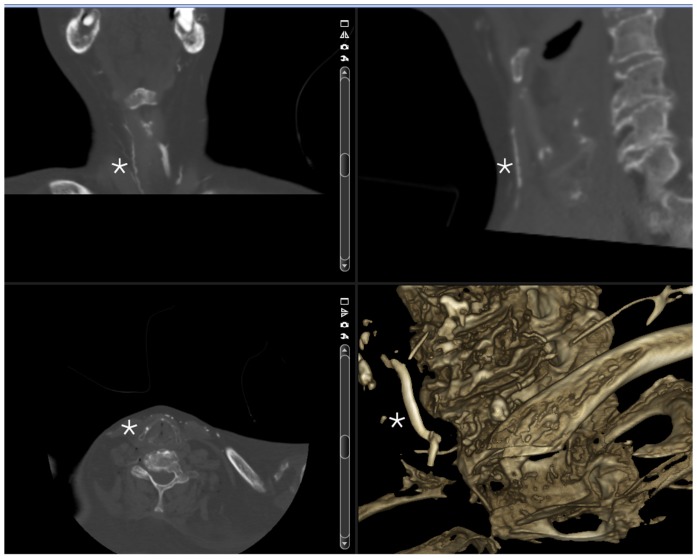
Multiplanar CT reconstruction and surface rendered image showing vessels (right to the asterisk) after barium sulfate injection into an intraosseous access placed into the left anterior mandible.

Figure 7 depicts the traces of barium sulphate injected into the left mandible between midline and the foramen mentale obtained in a CT scan of a separate test.

The puncture success rate observed in the group of the medical students was equal compared to the dental students and there were no differences between both groups regarding the time needed to perform the procedure. These student groups had been chosen as homogeneous populations regarding anatomy knowledge from the recent anatomy course experiences. The dental students were thought to have a closer affinity to the anatomy structures of the jaws. They were assumed to be more familiar with lower jaw clinical anatomy, as they were practicing local anesthesia – also in the area of the foramen mentale - on each other during respective courses. The results, however, did not reflect different time requirements to perform the procedure and the populations did not differ regarding complications through the punctures.

The current pilot study demonstrates that:

1. The intraosseous puncture of the mandibula by use of the EZ-IO system may serve as an alternative to a vascular access if other bones are not available
2. Floor of mouth extravasation may complicate the success of the procedure
3. Despite extravasation intravascular application of medication may still be possible
4. The success rate is lower than described in the sites evaluated in other studies
5. The time needed to perform a successful procedure is comparable to the time frames needed for other bone puncture procedures

According to the pertinent literature, success rates of an intraosseous access range between 100 and 60% [3]. The success rate via the mandibular route found in this evaluation was lower than the one observed in most other studies. A comparable success rate has been observed in paramedics performing a sternal intraosseous access [18]. Considering homogenous populations of unexperienced test persons in the present evaluation, the success rate may be different in skilled emergency physicians and medical staff who are experienced in intraosseous punctures of other sites [3], [19], [20]. However, the knowledge of the students regarding mandibular anatomy according to their recent dissecting course experiences may have been better than the average emergency physician knowledge of this area. The time needed to perform the mandibular intraosseous access is in the order of magnitude needed to puncture other sites [9], [18], [21].

The battery operated drilling device has been chosen to facilitate the expected high resistance of the cortical layer of the mandibular bone.

As expected, floor of mouth extravasation may be a significant problem, especially due to the close vicinity to the airway.

**Figure 8:**
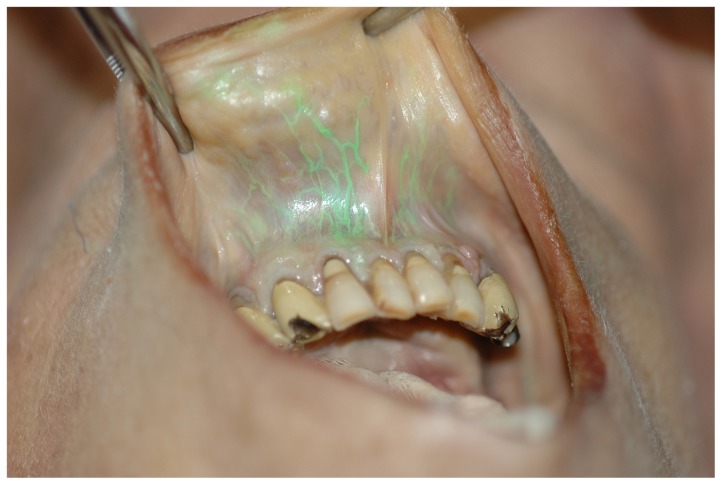
Vessels of the lower lip stained by injection of green dye into a mandibular intraosseous vascular access.

However, it can be shown in post mortem demonstrations of liquid dye, that successful intravascular administration through the mandible may be verified before administering medication. An example obtained in an independent experiment is shown in figure 8.

If floor of mouth extravasation is not detected, this can result in severe problems such as airway obstruction [22], especially when an infusion of a higher amount of fluid is administered. Extravasation of other medication in this area may also result in more severe complications than in other parts of the body.

To reduce the likelihood of undetected extraosseous needle placement, an aspiration test in living subjects would be recommended. A similar recommendation exists for other sites of intraosseous punctures. The reliability of an aspiration test may be restricted in case of limited medullary cavity space in the atrophic mandible. In case of intraosseous extramedullary position of the needle a successful application of medication may still be possible [23] even if the aspiration test has a negative result.

The use of dyes to verify a correct intraosseous cannula placement in the living tissue might also be an option. Intraosseous application of methylene blue has been described previously [24]. Subcutaneous extravasation of methylene blue may, however, later result in tissue necrosis [25]. Indocyanine green, which has been widely used even intravenously may serve as an alternative [26].

Epinephrine as an alternative may not be visually detectable depending on the actual perfusion of the mucosa in the respective emergency situation but monitoring hemodynamic changes might be possible. Intraosseous epinephrine exceeding 1∶100,000 [17], [27] should result in a detectable heart rate and or blood pressure increase – depending on the hemodynamic status [28].

During the test injection in the presented study, only a very low pressure was applied to avoid excessive ink extravasation in case of extraosseous cannula placement. As flush pressures, especially during testing injection of the cannula, may far exceed 2,500 mmHg [29], [30], special care would be advisable to avoid excessive extravasation e.g. into the floor of mouth tissue. The injection pressure was not measured in our study. But the pressure necessary for injection into the mandible was rated as within a range comparable to injections into the tibia, but higher, when compared to the manubrium sterni.

**Figure 9:**
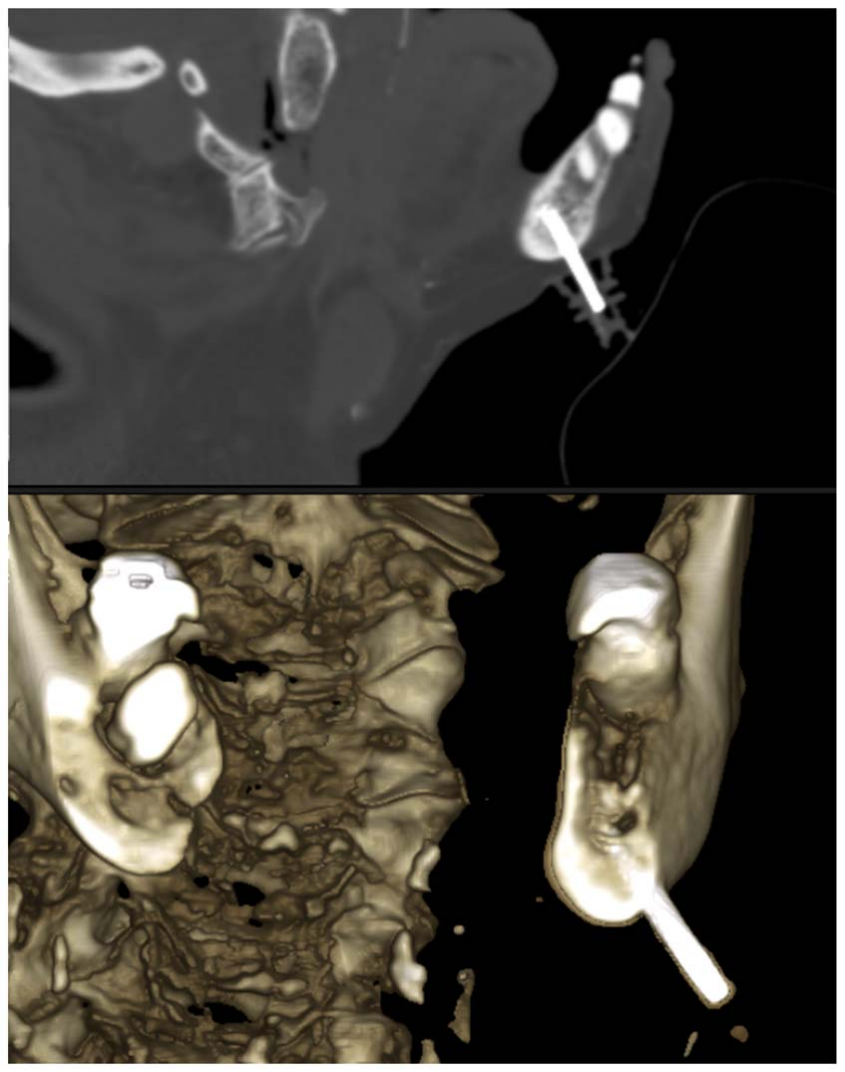
CT scan of a separate experiment is showing a successful puncture ending at the cortical layer of the left anterior mandible.

Increased pressures, however, may be observed in cases of a cannula tip position entering the opposite cortical layer of the mandible or when the cannula tip is positioned in the immediate vicinity of this area. Furthermore, the rate of punctures ending at or in the cortical layer of the mandible (a CT scan of a separate experiment is shown in figure 9) or even perforations may be higher compared to other sites recommended for intraosseous punctures, especially when unexperienced physicians/dentists would use this site. A higher injection/infusion pressure may be required if the cannula tip is located at the opposite cortical layer of the mandible. Aspiration of bone marrow as a marker of intraosseous position may be of limited value in this case as well.

### Limitations

The study has the typical limitations all examinations in cadavers have in contrast to living subjects such as fixated tissues and no existing circulation. Futhermore, the bone structures and relations of marrow and cortical bone of the body donors may not represent the average emergency patient being a possible candidate for such a puncture procedure. A bone marrow aspiration test to verify the correct needle placement was not possible in the cadavers, but may also be difficult or impossible in living humans [31]. A pre injection procedure was not chosen to prevent a dilution of ink as well as a higher volume of injection - as the cadavers were scheduled for the gross anatomy dissection course and especially paravascular staining could have resulted in severe discoloration of large tissue areas impairing the ability to identify anatomical structures for students and instructors. Even with the low volume of injected ink, large areas were stained in case of paravascular position of the cannula tip. The sample size was relatively small and the puncture was only performed once. It is not clear, if better success rates might be achieved in repeated trials [10] or when trained medical staff experienced in the puncture of other intraosseous access sites would perform the procedure. Flow rates which can be achieved by such a procedure during fluid resuscitation have also not been evaluated [32]. Extravasation as has been observed even with similar intravascular staining may complicate success of the procedure when pressure infusions are used. A repeated puncture in case of an obvious puncture failure may result in even enhanced fluid extravasation from the first puncture site, especially in cases of fluid resuscitation. Although no fracture of the jaw was observed in this evaluation, this complication may occur in cases of severe osteoporosis or in atrophic mandibles.

## Conclusions

The mandibular intraosseous access and infusion into the mandible may work and may be trained in a standardized way. Within the limitations of this study it can be stated that the mandibular intraosseous access may represent an alternative vascular route in desperate cases under extraordinary circumstances e.g. entrapment when other methods fail or are not available. Extravasation especially into the floor of mouth tissue may significantly influence the success of such a procedure and severe complications such as airway obstruction might result.

To familiarize with the procedure, repeated cadaver training should be included into educational sessions where first line access sites would be trained as well.

Further studies of the mandibular intraosseous access should involve the application of medication and fluid.

## Supporting Information

Video S1 Demonstration of intraosseous vascular access through the anterior mandible using a battery powered motor driven drilling device.
(WMV)
